# A Survey on the Criteria Used to Judge (Fake) News in Italian Population

**DOI:** 10.1002/brb3.70315

**Published:** 2025-02-11

**Authors:** Fabiana Battista, Tiziana Lanciano, Antonietta Curci

**Affiliations:** ^1^ Department of Education, Psychology, Communication University of Bari “Aldo Moro” Bari Italy

**Keywords:** deception detection, fake news, Italian population, veracity judgments, verbal criteria

## Abstract

**Introduction:**

Fake news detection falls within the field of deception detection and, consequently, can be problematic due to no consensus on which cues increase the detection accuracy and because people's ability to detect is poor.

**Methods:**

The present study aimed to investigate the criteria used by general population to establish if a given news item is true or fake by surveying a sample of the Italian population. We recruited 329 participants who had to reply to some questions on which criteria they used to conclude a given news item was true. The same questions were also asked to investigate the ones used for fake news judgments.

**Results and Conclusion:**

Our results showed that, overall, people use similar criteria (e.g., reliability of the source and presence of scientific references) to conclude that news is true versus fake, although their use rates differ for true and fake news.

## Graphical abstract

Criteria to detect fake versus truthful news in Italian population.

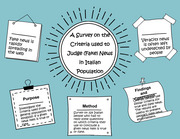

## 1 Introduction

In the last decades, the phenomenon of fake news has been gathering attention from researchers due to its massive spread over the World Wide Web (Greifeneder et al. 2021; Khan and Indris 2019; Lazer et al. 2018; Pennycock and Rand 2021; Porter 2020). As a matter of fact, although there is proof of fake news dissemination already in 1935 when the American newspaper The Sun published the so‐called Great Moon Hoax articles, an important increase of this phenomenon occurred recently, especially after 2016 with false information for the USA presidential election when on Facebook were shared politically oriented fake news as well as with the news related to the coronavirus disease 2019 (COVID‐19) (Loomba et al. 2021; van der Linden, Roozenbeek, and Compton 2020; Wardle 2018).

Fake news has been defined as any intentional fabricated information that imitates original information provided by the media in its structure, but which follows a different organizational process or intent and could mislead readers (Allcott and Gentzkow 2017; Lazer et al. 2018). Consequently, fake news include different forms of false information, namely manipulation, disinformation (i.e., news deliberately misleading and deceiving), misinformation (i.e., information that is verifiably false or inaccurate and intends to deceive its receiver that could spread it even unintentionally), rumors, and news satire and parody (Aoun Barakat, Dabbous, and Tarhini 2021; Lazer et al. 2018; Ruffo and Semeraro 2022; Tandoc, Lim, and Ling 2018). For instance, Tandoc, Lim, and Ling (2018) conducted a review to understand the categories of fake news identified by researchers over the years. They found that, in the 34 articles reviewed, previous studies have operationalized fake news in 6 categories, namely, satire (i.e., false news programs with humor or exaggeration), parody (i.e., false news using non‐factual information to make humor), fabrication (i.e., articles with no factual basis but highly resembling true news), photo manipulation (i.e., manipulation of real images or videos to create a false narrative), propaganda (i.e., news stories created for a political entity to influence public perceptions), and advertising (i.e., false advertisements included in true news or advertisements referring to information not really included in the article, i.e., clickbait).

Some studies have tried to understand the exact prevalence of this phenomenon throughout the web (e.g., Allcot and Gentzkow 2017; Grinberg et al. 2019; Guess, Nagler, and Tucker 2019; Rogers 2020). The main method used to make this estimation consisted in looking at how often people reposted and shared fake news articles, specifically information on USA election on their social media. Overall, they found a low sharing of fake news, thus arguing that fake news prevalence might be lower than expected. However, these data do not properly provide a measure of fake news frequency as they actually provide information on people's tendency to share fake information. Indeed, Vosoughi, Roy, and Aral (2018) supported the idea that fake news prevalence increases robustly especially in the last years due to the wide use of individuals of the web (e.g., Lazer et al. 2018; Pennycock and Rand 2021; Vosoughi, Roy, and Aral 2018). They investigated the prevalence of true and false news on Twitter by checking around 126,000 news spread by 3 million people and by considering also additional measures, such as how fast news spread on the web and the content of these news. They discovered that the frequency of false news was higher and they are diffused faster than true ones. In addition, they found that their content was related to different topics, such as politics, business, terrorism, science, entertainment, and natural disasters.

As a matter of fact, beyond false political information, fake news might also concern any other topic, such as, for instance, medical issues (Carrieri, Madio, and Principe 2019; Chen, Wang, and Peng 2018; Lyons, Merola, and Reifler 2019) and climate changes (Lopez and Share 2020; Lutzke et al. 2019). This occurs because, as Ahmed (2017) suggested, everyone can easily write fake news on the web. In addition, nowadays there are several webpages purposely developed to publish fake news and information (e.g., denverguardian.com, wtoe5news.com, and ABCnews.com.co). Zhang and Ghorbani (2020) identified that authors of fake news can be either humans or nonhumans. Indeed, humans can disseminate fake news by directly writing and publishing false content or by programming automatic and malicious accounts (e.g., social bots and cyborgs) to do so. In both cases, in order to seem credible, fake news creation relies on true and/or scientific information, becoming difficult to spot due to the similarities with true news in terms of content and linguistic features (Shu et al. 2017). In addition, Voloch, Gudes, and Gal‐Oz (2021) underlined that fake news propagation depends not only on people with malicious intentions but also because of people having low social media awareness spreading online fake information. The authors studied the activities of these people to understand how they foment fake news propagation with the final aim to use machine learning to detect and prevent these activities and prevent them.

Moreover, oftentimes fake news aligns with individuals’ ideology, beliefs, and knowledge by making them believable (Bryanov and Vziatysheva 2021; Lewandowsky et al. 2019; Moravec, Minas, and Dennis 2018; Pereira and Van Bavel 2018). For instance, Pereira and van Bavel (2018) conducted three experiments to test the (i) ideology, (ii) confirmation bias, and (iii) political identity hypotheses, namely, individuals’ preference to news that aligns (i) their values, (ii) their prior knowledge, and (iii) their political ideology. Specifically, they asked participants to respond to some questions concerning their political orientation. Then, they were randomly assigned to one of the two conditions (political congruent news vs. political noncongruent news) and asked to read seven news (fake and true) related either to Hillary Clinton or Donald Trump or values (i.e., liberal vs. conservative) supported by one of the two political characters. Hence, participants were asked to rate how much they believed in such news. In all three experiments, the authors found a robust effect of people's ideology and knowledge in their propensity to believe in fake news, and this effect did not differ in liberal and conservative people. In 2019, these results were also shown by Anthony and Moulding (2019). In their study, the authors tried to look also at other individuals’ characteristics that can affect people likelihood to believe in fake news. In particular, beyond politically congruent beliefs, they verified the association among conspiratorial beliefs, schizotypal personality, and fake news and found that both politically congruent and conspiratorial beliefs predicted beliefs in fake news.

Overall, scholars have carried out numerous studies to unveil the characteristics of people falling for fake news. Several cognitive and personality individuals’ factors have been identified (Bronstein et al. 2019; Calvillo et al. 2021; Pennycook and Rand 2020; Sindermann, Cooper, and Montag 2020; Taurino et al. 2023; Wolverton and Stevens, 2020; for a review see Bryanov and Vziatysheva 2021). In terms of cognitive factors, analytical thinking (i.e., the process compromising deliberation, contemplation, and logically reasoning; Evans and Stanovich 2013) seems to be the most relevant for fake news detection (Bronstein et al. 2019; Pennycook and Rand 2020; Taurino et al. 2023). Pennycook and Rand (2020) showed that analytical thinking is indeed informative of people proneness to discern fake news from real news such that people higher in analytical thinking were more able to detect fake news. Subsequently, this result was widely replicated in several studies (Bago, Rand, and Pennycook 2020; Bronstein et al. 2019; Machete and Turpin 2020; Martel, Pennycook, and Rand 2020; Pennycook and Rand 2020).

In terms of personality traits, some studies have shown that conscientiousness and openness to experience are negatively correlated with fake news accuracy detection, such that people higher in conscientiousness and openness traits were less able to correctly identify fake news (Sindermann, Cooper, and Montag 2020; Wolverton & Stevens, 2020). In addition, Calvillo et al. (2021) identified that agreeableness, conscientiousness, open‐mindedness, and extraversion are associated to individuals’ fake news detection, with a positive correlation between agreeableness, conscientiousness, open‐mindedness, and accuracy detection and a negative association with extraversion.

Irrespective of the individuals’ characteristics that can influence people's tendency to spot fake news, fake news detection is particularly difficult also because oftentimes this news is emotionally captivating (e.g., Voloch, Petrocchi, and De Nicola 2024; Vosoughi, Roy, and Aral 2018) or is repeatedly encountered by people due to the widespread overall the web (Flaxman, Goel, and Rao 2016).

### 1.1 Fake News and Deception Detection

Fake news identification and detection belong to the broader concept of deception detection, and consequently, evidence coming from this line of research can be applied to the specific context of false news. The prominent and recurrent finding of deception detection studies is that people ability to discriminate the truth from lies is around 54% (Bond and DePaulo 2006). This occurs because people are prone to different cognitive biases in the evaluation of the accuracy of a statement (e.g., Levine, Park, and McCornack 1999; 2014; McCornack and Parks 1986; Park and Levine 2017; Zuckerman, DePaulo, and Rosenthal 1981). The most known are the *truth‐bias*, according to which individuals believe that people overall convey truthful information (Bond and DePaulo 2006; Levine, Park, and McCornack 1999; McCornack and Parks 1986; Park and Levine 2017; Zuckerman, DePaulo, and Rosenthal 1981) and the *default‐bias*, an automatic inclination to passively accept as honest others’ statements. Levine (2014) built up a theory based on these two biases, the so‐called truth‐default theory (TDT) clarifying that these two biases differ from each other because of the involvement of the person evaluating the statement during the inferring process. He noted that the truth‐bias occurs due to an active judgment of the veracity of the statement, whereas the truth‐default because of a passive judgment. As a consequence to these kinds of biases, people also have a tendency to be more accurate in their detection of truthful statements than of false statements, namely, the *veracity effect* (Levine, Park, and McCornack 1999). Similar to this type of biases, Peace and Sinclair (2012) identified the *emotional truth‐bias*, which is the individuals’ likelihood to detect as true emotional false events because of the emotional content of statements. In addition, research has also shown another interesting effect due to the repeated exposure to the same information, the *illusory truth effect*, such that when people have a proneness to evaluate as true statements already seen instead of new ones (Hasher, Goldstein, and Toppino 1977; Newman et al. 2020).

Scholars in the field of deception detection have run a plethora of studies to understand whether deception detection can be improved by adopting specific cues, such as behavioral, physiological, and verbal cues (e.g., Battista et al. 2024; Curci et al. 2019; for a meta‐analysis see Hartwig and Bond 2014). So far, scholars propend to believe that verbal cues are the most helpful although—even by using these cues—the highest accuracy rate reached is around 70% (e.g., Hartwig and Bond 2014). On the basis of this evidence, several techniques to detect deception have been developed. The criteria‐based content analysis (CBCA; Köhnken 2004), the reality monitoring (RM; Johnson and Raye 1981), the scientific content analysis (SCAN; Smith and Willis, 2001), and the verifiability approach (VA; Nahari, Vrij, and Fisher 2012) are just some of them.

Specifically, the CBCA (Köhnken 2004) is a part of the statement validity analysis and consists of the analysis of the presence (or absence) of nineteen criteria clustered in four areas, that is, general characteristics, specific contents, peculiarity of the content, and motivational reasons. Specifically, the general characteristics include possible inconsistencies, how the information is presented, and the quantity of details reported. The specific contents category corresponds to the type of details reported (i.e., contextual, unusual, superfluous, and event‐specific), whether possible conversations and interactions between people are reported as well as id feelings and thoughts experienced at the time of the event that are included. The motivational reasons category takes into account spontaneous corrections, lack of memory, and doubts about one's own statement. It is assumed that when statements are true, these criteria are met to a higher extent than when statements are false.

Similarly, the RM (Johnson and Raye 1981) is based on the idea that truthful and deceptive information differ in terms of perceptual and contextual information present. The underlying assumption here is that truthful statement usually contains very detailed and clear information such as perceptual (e.g., sounds, smell, and visual details) and contextual—both spatial and temporal—one (i.e., where the event took place and people involved in the event), whereas false information usually are vague and consists of cognitive operations (i.e., thoughts and reasoning).

The SCAN (Smith and Willis, 2001) relies on a list of 12 criteria, some of them more likely to occur in truthful statements than in deceptive statements (e.g., the use of pronouns), whereas others more likely to occur in deceptive statements than in truthful statements (e.g., missing information). Precisely, these criteria are as follows: denial of allegations, social introduction, spontaneous corrections, lack of conviction and memory, structure of statement, emotions, objective and subjective time, out‐of‐sequence and extraneous information, missing information, first person singular past tense, pronouns, and change in language.

Finally, the VA (Nahari, Vrij, and Fisher 2012) is a verbal technique based on two arguments: (i) Liars prefer to provide many details, because detailed accounts are more likely to be believed, (ii) liars prefer to avoid mentioning too many details that can be verified. Therefore, false statements report many details that are verifiable. Hence, false statements are longer and composed of not‐checkable details as compared to true statements.

These techniques have been mainly used in forensic context to discern truthful and deceitful statements concerning crimes, and the majority of studies have focused attention on the false versus true statements detection in this regard (e.g., Curci et al. 2019). However, some of these criteria are also relevant for fake news detection, and it is reasonable to believe they are also the ones used by individuals to conclude whether they are dealing with a true or fake news (i.e., fake vs. true news judgments). One attempt to investigate the criteria used to recognize fake news was made by Weiss et al. (2020). Their survey had the specific aim to understand how a group of academics (e.g., professors, assistant professors, and lecturers) teaching in different faculties of an American university define fake news and recognize them (e.g., based on intuition, the origin of the news, by fact‐checking). They found that the criteria used to recognize news as fake varied based on the faculties and rank of academics. These results underline that there is a lack of concordance even among people having a high education (i.e., academics). Therefore, understanding the criteria adopted by the general public (i.e., individuals who daily deal with news and have no specific degree or education on the matter) to establish if news are false or true is important to educate people on this matter. Indeed, the problems associated with endorsing fake news are numerous (Cantarella, Fraccaroli, and Volpe 2023; Green and Murphy 2020; Mangiulli et al. 2022; Murphy et al. 2019; Riedel et al. 2017). Studies have shown that fake news detrimentally affect people's behaviors (Balakrishnan et al. 2023; Rapp and Salovich 2018), for instance, by increasing political and partisan conflict as well as by influencing voters (Cantarella, Fraccaroli, and Volpe 2023; Riedel et al. 2017), by affecting people's memory and beliefs about the occurrence of the fake news (Green and Murphy 2020; Mangiulli et al. 2022; Murphy et al. 2019).

### 1.2 The Present Study

We aimed to investigate how (i.e., which criteria are used) the general population (i.e., no professionals or experts) judge news as true versus fake (Aim 1) and if there are differences in how the conclusion is reached between true and fake news (Aim 2). Precisely, we surveyed a sample of Italian population concerning the criteria reported to be adopted to judge news as truthful or deceitful. We first asked participants to provide their demographic information (i.e., age, gender, and education) and then to reply to some questions regarding the specific criteria used to judge the authenticity of the news, how these criteria change based on where the news is published, and the reasons why people use such criteria. Participants had to reply to these questions first by thinking about what leads them to believe the news are true and then about what makes them believe the news are false. The construction of the survey (i.e., definition of the questions and criteria) was driven by the above‐described literature on deception detection and the criteria constituting deception detection tools (e.g., CBCA, Köhnken 2004; RM, Johnson and Raye 1981; SCAN, Smith and Willis, 2001; VA, Nahari, Vrij, and Fisher 2012) but also by additional criteria that could represent the specificities of true versus fake news detection, such as the reliability of the source, the sharing of the news by other people, or fact‐checking. These additional criteria are also in line with the ones proposed in prior research (e.g., Weiss et al. 2020). In addition, we decided to target the general population with Italian nationality because, to the best of our knowledge, so far no study has collected information on this population.

Because of the lack of studies aiming to understand the criteria adopted to conclude about the truthfulness (or not) of news and possible differences among these criteria, we were also unable to draw specific hypotheses.

Before starting the study, we received the ethical approval from the Ethical Board of the *blind* Department of Education, Psychology, Communication of the University of Bari Aldo Moro (ET‐23‐08). In addition, before starting data collection, the study was preregistered on Open Science Framework: *blind* https://osf.io/r2kdf/.

## 2 Methods

### 2.1 Sample

A sample of 383 participants was recruited but only 329 people (women = 71.7%, *M*_age_ = 29.93, SD = 12.42) completed the survey which was conducted online by using Qualtrics platform. The sample was recruited on a voluntary basis by advertisements on social media, psychology students of the University of Bari Aldo Moro, and through word of mouth and no compensation or course credit was granted. All participants signed the informed consent before undertaking the study, and anonymity was guaranteed to all participants.

### 2.2 Procedure and Measures

Before starting the survey, all participants read and signed the informed consent. Immediately after, participants filled out the survey which was constructed ad hoc by the authors with multiple choice options.

We first collected participants’ demographic information (i.e., age, gender, and education) and presented them with a small description of what are fake news in order to assure that they properly replied to our questions. Then, participants had to complete the questionnaire, which was composed of two main sections, completely identical except for the instructions: The first section was on think of true news judgments and the second section on fake news judgments. Each section was composed of the three questions.

For both sections, participants replied to the questions by thinking of what criteria they use to judge the (un)truthfulness of the news encountered and for which they do not have any information in this regard before reading or listening to them. In other words, participants had to report the criteria that make them conclude the news are true (Section 1) or fake (Section 2). For Question 1 concerning the criteria adopted to judge news, participants might select more than one criterium among the ones listed. The criteria were as follows: (i) consistency of the content, (ii) clarity of the content, (iii) amount of details, (iv) vividness of details, (v) type of details reported, (vi) presence of unusual details and superfluous details, (vii) context‐related information, (viii) description of interactions, (ix) presence of conversations, (x) external associations, (xi) description of the subjective mental state of the characters in the news, and (xii) verifiability of the information, (xiii) presence of references to scientific studies, (xiv) reliability of the source, (xv) emotions aroused when reading/listening to the news, (xvi) sharing of the news by other people, and (xvii) confirmation of the news by searching for it in other sources. For Question 2, participants had to indicate whether they used the selected criteria for different sources of the news (i.e., television, newspapers, websites, and social media). If they replied yes, participants might reply to the third and last question of the section. Otherwise, for each of the sources of information (i.e., television, newspapers, websites, and social media), they had to select one or more of the criteria presented in the first question. With Question 3, we aimed to collect information on the reasons why people use the above‐selected criteria among the options: (i) based on my prior experience, (ii) because I am informed concerning the criteria that can help me in discriminating true versus fake news, and (iii) I do not know. Finally, if participants selected option (ii), they had to respond to a follow‐up question regarding how they achieved such a knowledge (e.g., scientific papers and books).

After filling both sections, participants were thanked and debriefed concerning the scope of the study.

## 3 Results

Results are presented in three sections: A first section focused on criteria used for true news, and a second section on data on false news. The third section provides a comparison of these criteria. For each section, we report the first five criteria selected, and complete data of each section are provided in tables (Table 1 for true news, Table 2 for fake news, and Table 3 for their comparison).

### 3.1 True News

**FIGURE 1:**
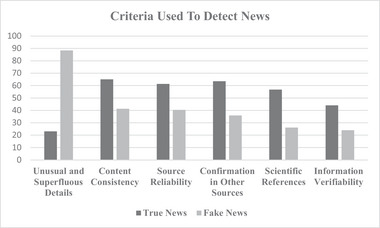
This figure shows the most used criteria adopted to identify a news as true or fake.

*Question 1 (i.e., criteria used to judge a news as true)*. Overall, 65.0% (*n* = 214) of participants reported to adopt consistency of the content, 63.5% (*n* = 209) of participants indicated the confirmation of the news by searching for it in other sources, 61.4% (*n* = 202) considered the reliability of the source, 56.8% (*n* = 187) considered presence of references to scientific studies, and 44.1% (*n* = 145) the verifiability of the information (see also Figure 1).

*Question 2 (i.e., use of criteria for different sources)*. Overall, 86.6% (*n* = 285) referred to use the same criteria, whereas 11.9% (*n* = 39) reported to use different criteria based on the source of information. Regarding television, 71.8% (*n* = 28) of participants referred to the consistency of the content, 43.6% (*n* = 17) the clarity of the content, 30.8% (*n* = 12) the presence of references to scientific studies and the reliability of the source, and finally, 25.6% (*n* = 10) the type of details reported and context‐related information. Concerning newspapers, 61.85% (*n* = 24) of participants reported the consistency of the content, 48.7% (*n* = 19) the clarity of the content, 33.3% (*n* = 13) the amount of details and the reliability of the source, and 28.2% (*n* = 11) of participants the confirmation of the news by searching for it in other sources. With regard to websites, 71.8% (*n* = 28) of participants reported the consistency of the content, 51.3% (*n* = 20) the reliability of the source, 48.7% (*n* = 19) the clarity of the content, 46.1% (*n* = 18) the presence of references to scientific studies, and 35.9% (*n* = 14) the verifiability of the information. Finally, concerning social media, 64.1% (*n* = 25) reported that they consider the consistency of the content, 51.3% (*n* = 20) the reliability of the source, 41.0% (*n* = 16) the presence of references to scientific studies, 35.9% (*n* = 14) clarity of the content, confirmation of the news by searching for it in other sources, and the verifiability of the information.

*Question 3 (i.e., reasons why they adopt the selected criteria)*. Overall, 47.1% (*n* = 155) reported to base their judgments in accordance with their prior experience, 45.6% (*n* = 150) because they are informed concerning the criteria that can help them in discriminating true versus fake news, and 7.3% (*n* = 24) do not have specific reasons (i.e., “I do not know”). People who reported of being informed referred to have use the following sources: 50.0% (*n* = 75) scientific articles, 20.0% (*n* = 45) television, 36.6% (*n* = 55) books, 65.3% (*n* = 98) websites, and 36.0% (*n* = 54) social media.

### 3.2 Fake News

*Question 1 (i.e., criteria used to judge a news as fake)*. Overall, 88.4% (*n* = 291) of participants referred to use the presence of unusual and superfluous details, 41.3% (*n* = 136) reported to adopt the consistency of the content, 40.4% (*n* = 133) referred to adopt the reliability of the source, 35.9% (*n* = 118) the criterium of confirmation of the news by searching for it in other sources, and 26.1% (*n* = 86) of participants both the type of details reported and the presence of references to scientific studies (see also Figure 1).

*Question 2 (i.e., use of criteria for different sources)*. Overall, 92.4% (*n* = 304) referred to use the same criteria for all types of sources of information, whereas 7.6% (*n* = 25) did not. As for true news, those last group of individuals referred to the different criteria for each source of information. With regard to television, 52.0% (*n* = 13) of participants reported the consistency of the content, 36.0% (*n* = 9) the clarity of the content, 32.0% (*n* = 8) the context‐related information, 28.0% (*n* = 7) the reliability of the source, and 24.0% (*n* = 6) the criterium of confirmation of the news by searching for it in other sources. Regarding newspapers, 60.0% (*n* = 15) of participants reported the consistency of the content, 48.0% (*n* = 12) the clarity of the content, 40.0% (*n* = 10) the presence of references to scientific studies, 28.0% (*n* = 7) of participants the reliability of the source, and 25.0% (*n* = 7) the amount of details and the vividness of the source. Concerning websites, 52.0% (*n* = 13) of people referred to the consistency of the content, 44.0% (*n* = 11) the reliability of the source, 36.0% (*n* = 9) the clarity of the content, 28.0% (*n* = 7) the presence of unusual and superfluous details, the presence of references to scientific studies, and the criterium of confirmation of the news by searching for it in other sources. Regarding social media, 52.0% (*n* = 13) considered the consistency of the content, 40.0% (*n* = 10) the reliability of the source, 36.0% (*n* = 9) the presence of references to scientific studies, the confirmation of the news by searching for it in other sources, and the clarity of the content.

*Question 3 (i.e., reasons why they adopt the selected criteria)*. Overall, 52.9% (*n* = 174) referred to base their judgments on their prior experience, 39.2% (*n* = 129) because they informed themselves concerning the criteria that can help me in discriminating true versus fake news, and 7.9% (*n* = 26) with no specific reasons (i.e., “I do not know”). People who referred to being educated specify that they have this information because of the following sources: 53.5% (*n* = 69) scientific articles, 31.0% (*n* = 40) television, 41.9% (*n* = 54) books, 67.4% (*n* = 87) websites, and 37.2% (*n* = 48) social media.

### 3.3 Comparison of Criteria

*Question 1 (i.e., criteria used to judge a news)*. We ran comparison analyses on frequencies reported to each criterion for true versus fake news to check for statistically significant differences. Specifically, we found statistically significant differences in the use of the following criteria: The consistency of the content is adopted differently for true and fake news, *χ* ^2^ (1, *N* = 349) = 17.4, *p* < 0.001, Cohen's *W* = 0.22, clarity of the content, *χ* ^2^ (1, *N* = 218) = 13.8, *p* < 0.001, Cohen's *W* = 0.20, amount of details, *χ* ^2^ (1, *N* = 168) = 5.69, *p* = 0.02, Cohen's *W* = 0.13, presence of unusual details and superfluous details, *χ* ^2^ (1, *N* = 214) = 53.3, *p* < 0.001, Cohen's *W* = 0.40, context‐related information, *χ* ^2^ (1, *N* = 147) = 16.9, *p* < 0.001, Cohen's *W* = 0.23, presence of conversations, *χ* ^2^ (1, *N* = 92) = 11.7, *p* < 0.001, Cohen's *W* = 0.19, description of the subjective mental state of the characters in the news, *χ* ^2^ (1, *N* = 85) = 7.86, *p* = 0.005, Cohen's *W* = 0.15, verifiability of the information, *χ* ^2^ (1, *N* = 272) = 37.4, *p* < 0.001, Cohen's *W* = 0.34, presence of references to scientific studies, *χ* ^2^ (1, *N* = 224) = 19.4, *p* < 0.001, Cohen's *W* = 0.24, reliability of the source, *χ* ^2^ (1, *N* = 334) = 14.2, *p* < 0.001, Cohen's *W* = 0.21, emotions aroused when reading/listening to the news, *χ* ^2^ (1, *N* = 69) = 8.23, *p* = 0.004, Cohen's *W* = 0.16, sharing of the news by other people, *χ* ^2^ (1, *N* = 120) = 5.17, *p* = 0.02, Cohen's *W* = 0.13, confirmation of the news by searching for it in other sources, *χ* ^2^ (1, *N* = 326) = 25.3, *p* < 0.001, Cohen's *W* = 0.28. Precisely, participants reported to use the criteria more for true news than fake news (i.e., emotions aroused when reading/listening to the news and sharing of the news by other people) for which we found an opposite pattern. By contrast, no significant differences were found for the criteria: Vividness of details, *χ* ^2^ (1, *N* = 74) = 0.65, *p* = 0.42, Cohen's *W* = 0.04, type of details reported, *χ* ^2^ (1, *N* = 190) = 1.89, *p* = 0.17, Cohen's *W* = 0.08, description of interactions, *χ* ^2^ (1, *N* = 57) = 1.72, *p* = 0.19, Cohen's *W* = 0.07, external associations, *χ* ^2^ (1, *N* = 85) = 3.11, *p* = 0.08, Cohen's *W* = 0.10, and others, *χ* ^2^ (1, *N* = 8) = 0.11, *p* = 0.74, Cohen's *W* = 0.02.

*Question 2 (i.e., use of criteria for different sources)*. We did not find a statistically significant difference between true and fake news, *χ* ^2^ (1, *N* = 657) = 6.01, *p* = 0.05, Cramer's *V* = 0.10.

*Question 3 (i.e., reasons why they adopt the selected criteria)*. Participants did not report any statistically significant differences for true and fake news with regards to all reasons, specifically (i) reliance on their prior experience, *χ* ^2^ (1, *N* = 143) = 0.25, *p* = 0.62, Cohen's *W* = 0.02 (ii) informing themselves on the criteria that can help me in discriminating true versus fake news, *χ* ^2^ (1, *N* = 84) = 0.29, *p* = 0.59, Cohen's *W* = 0.04, and (iii) no specific reasons (i.e., “I do not know”), *χ* ^2^ (1, *N* = 29) = 0.13, *p* = 0.72, Cohen's *W* = 0.02.

**TABLE 1:** This table shows the percentages of participants’ responses to questions concerning true news.

Question	Option	Percentage			
Which criteria do you use to conclude a news is true?	Consistency of the content	65.0 (*n* = 214)			
	Clarity of the content	41.6 (*n* = 137)			
	Amount of details	30.4 (*n* = 100)			
	Vividness of details	12.5 (*n* = 41)			
	Type of details reported	31.9 (*n* = 105)			
	Presence of unusual details and superfluous details	23.1 (*n* = 76)			
	Context‐related information	30.1 (*n* = 99)			
	Description of interactions	10.3 (*n* = 34)			
	Presence of conversations	23.4 (*n* = 77)			
	External associations	11.6 (*n* = 38)			
	Description of the subjective mental state of the characters in the news	9.1 (*n* = 30)			
	Verifiability of the information	44.1 (*n* = 145)			
	Presence of references to scientific studies	56.8 (*n* = 187)			
	Reliability of the source	61.4 (*n* = 202)			
	Emotions aroused when reading/listening to the news	7.0 (*n* = 23)			
	Sharing of the news by other people	14.6 (*n* = 48)			
	Confirmation of the news by searching for it in other sources	63.5 (*n* = 209)			
	Others	1.2 (*n* = 4)			
Do you use the same criteria for different sources of information?	Yes	86.6 (*n* = 285)			
	No	11.9 (*n* = 39)			
If no, which criteria do you use to conclude a news is true when you use:		**Television**	**Newspapers**	**Websites**	**Social Media**
	Consistency of the content	71.8 (*n* = 28)	61.5 (*n* = 24)	71.8 (*n* = 28)	64.1 (*n* = 25)
	Clarity of the content	43.6 (*n* = 17)	48.7 (*n* = 19)	48.7 (*n* = 19)	35.9 (*n* = 14)
	Amount of details	20.5 (*n* = 8)	33.3 (*n* = 13)	28.2 (*n* = 11)	17.9 (*n* = 7)
	Vividness of details	10.3 (*n* = 4)	5.1 (*n* = 2)	5.1 (*n* = 2)	10.2 (*n* = 4)
	Type of details reported	25.6 (*n* = 10)	17.9 (*n* = 7)	25.6 (*n* = 10)	17.9 (*n* = 7)
	Presence of unusual details and superfluous details	23.1 (*n* = 9)	17.9 (*n* = 7)	28.2 (*n* = 11)	33.3 (*n* = 13)
	Context‐related information	25.6 (*n* = 10)	12.8 (*n* = 5)	12.8 (*n* = 5)	15.4 (*n* = 6)
	Description of interactions	5.1 (*n* = 2)	5.1 (*n* = 2)	2.6 (*n* = 1)	5.1 (*n* = 2)
	Presence of conversations	15.4 (*n* = 6)	10.2 (*n* = 4)	10.2 (*n* = 4)	—
	External associations	23.1 (*n* = 9)	23.1 (*n* = 6)	7.7 (*n* = 3)	10.2 (*n* = 4)
	Description of the subjective mental state of the characters in the news	12.8 (*n* = 5)	10.2 (*n* = 4)	—	—
	Verifiability of the information	20.5 (*n* = 8)	20.5 (*n* = 8)	35.9 (*n* = 14)	35.9 (*n* = 14)
	Presence of references to scientific studies	30.8 (*n* = 12)	23.1 (*n* = 9)	46.1 (*n* = 18)	41.0 (*n* = 16)
	Reliability of the source	30.8 (*n* = 12)	33.3 (*n* = 13)	51.3 (*n* = 20)	51.3 (*n* = 20)
	Emotions aroused when reading/listening to the news	—	5.1 (*n* = 2)	10.2 (*n* = )	7.7 (*n* = 3)
	Sharing of the news by other people	—	—	17.9 (*n* = 7)	20.5 (*n* = 8)
	Confirmation of the news by searching for it in other sources	15.4 (*n* = 6)	28.2 (*n* = 11)	30.8 (*n* = 12)	35.9 (*n* = 14)
	Others	2.6 (*n* = 1)	5.1 (*n* = 2)	—	—
Why do you use those criteria?	Based on my experience	47.1 (*n* = 155)			
	I am informed concerning the criteria that can help me in discriminating true versus fake news because of:	45.6 (*n* = 150)			
	1. Scientific articles	50.0 (*n* = 75)			
	2. Television	20.0 (*n* = 45)			
	3. Books	36.6 (*n* = 55)			
	4. Websites	65.3 (*n* = 98)			
	5. Social media	36.0 (*n* = 54)			
	I do not know	7.3 (*n* = 24)			

**TABLE 2:** This table shows the percentages of participants’ responses to questions concerning fake news.

Question	Option	Percentage			
Which criteria do you use to conclude a news is fake?	Consistency of the content	41.3 (*n* = 136)			
	Clarity of the content	24.9 (*n* = 82)			
	Amount of details	21.0 (*n* = 69)			
	Vividness of details	10.3 (*n* = 34)			
	Type of details reported	26.1 (*n* = 86)			
	Presence of unusual details and superfluous details	88.4 (*n* = 291)			
	Context‐related information	14.9 (*n* = 49)			
	Description of interactions	7.3 (*n* = 24)			
	Presence of conversations	12.2 (*n* = 40)			
	External associations	16.7 (*n* = 55)			
	Description of the subjective mental state of the characters in the news	17.0 (*n* = 56)			
	Verifiability of the information	24.0 (*n* = 79)			
	Presence of references to scientific studies	26.1 (*n* = 86)			
	Reliability of the source	40.4 (*n* = 133)			
	Emotions aroused when reading/listening to the news	14.3 (*n* = 47)			
	Sharing of the news by other people	22.2 (*n* = 73)			
	Confirmation of the news by searching for it in other sources	35.9 (*n* = 118)			
	Others	1.5 (*n* = 5)			
Do you use the same criteria for different sources of information?	Yes	92.4 (*n* = 304)			
	No	7.6 (*n* = 25)			
If no, which criteria do you use to conclude a news is true when you use:		**Television**	**Newspapers**	**Websites**	**Social Media**
	Consistency of the content	52.0 (*n* = 13)	60.0 (*n* = 15)	52.0 (*n* = 13)	52.0 (*n* = 13)
	Clarity of the content	36.0 (*n* = 9)	48.0 (*n* = 12)	36.0 (*n* = 9)	36.0 (*n* = 9)
	Amount of details	8.0 (*n* = 2)	20.0 (*n* = 5)	16.0 (*n* = 4)	20.0 (*n* = 5)
	Vividness of details	16.0 (*n* = 4)	20.0 (*n* = 5)	12.0 (*n* = 3)	12.0 (*n* = 3)
	Type of details reported	8.0 (*n* = 2)	12.0 (*n* = 3)	16.0 (*n* = 4)	32.0 (*n* = 8)
	Presence of unusual details and superfluous details	12.0 (*n* = 3)	16.0 (*n* = 4)	28.0 (*n* = 7)	24.0 (*n* = 6)
	Context‐related information	32.0 (*n* = 8)	8.0 (*n* = 2)	20.0 (*n* = 5)	8.0 (*n* = 2)
	Description of interactions	8.0 (*n* = 2)	8.0 (*n* = 2)	—	4.0 (*n* = 1)
	Presence of conversations	16.0 (*n* = 4)	—	8.0 (*n* = 2)	8.0 (*n* = 2)
	External associations	4.0 (*n* = 1)	4.0 (*n* = 1)	—	4.0 (*n* = 1)
	Description of the subjective mental state of the characters in the news	12.0 (*n* = 3)	16.0 (*n* = 4)	4.0 (*n* = 1)	—
	Verifiability of the information	16.0 (*n* = 4)	12.0 (*n* = 3)	20.0 (*n* = 5)	20.0 (*n* = 5)
	Presence of references to scientific studies	12.0 (*n* = 3)	40.0 (*n* = 10)	28.0 (*n* = 7)	36.0 (*n* = 9)
	Reliability of the source	28.0 (*n* = 7)	28.0 (*n* = 7)	44.0 (*n* = 11)	40.0 (*n* = 10)
	Emotions aroused when reading/listening to the news	—	—	—	—
	Sharing of the news by other people	—	—	24.0 (*n* = 6)	24.0 (*n* = 6)
	Confirmation of the news by searching for it in other sources	24.0 (*n* = 6)	12.0 (*n* = 3)	28.0 (*n* = 7)	36.0 (*n* = 9)
	Others	—	—	—	—
Why do you use those criteria?	Based on my experience	52.9 (*n* = 174)			
	I am informed concerning the criteria that can help me in discriminating true versus fake news because of:	39.2 (*n* = 129)			
	1. Scientific articles	53.5 (*n* = 69)			
	2. Television	31.0 (*n* = 40)			
	3. Books	41.9 (*n* = 54)			
	4. Websites	67.4 (*n* = 87)			
	5. Social media	37.2 (*n* = 48)			
	I do not know	7.9 (*n* = 26)			

**TABLE 3:** This table shows the statistical significance of the differences between the responses for true and fake news.

Question	Option	Significance between true and fake news	Direction
Which criteria do you use to conclude a news is true?	Consistency of the content	Yes	True > Fake
	Clarity of the content	Yes	True > Fake
	Amount of details	Yes	True > Fake
	Vividness of details	No	—
	Type of details reported	No	—
	Presence of unusual details and superfluous details	Yes	True > Fake
	Context‐related information	Yes	True > Fake
	Description of interactions	No	—
	Presence of conversations	Yes	True > Fake
	External associations	No	—
	Description of the subjective mental state of the characters in the news	Yes	True > Fake
	Verifiability of the information	Yes	True > Fake
	Presence of references to scientific studies	Yes	True > Fake
	Reliability of the source	Yes	True > Fake
	Emotions aroused when reading/listening to the news	Yes	True < Fake
	Sharing of the news by other people	Yes	True < Fake
	Confirmation of the news by searching for it in other sources	Yes	True > Fake
	Others	No	—
Do you use the same criteria for different sources of information?	Yes	No	—
	No	No	—
Why do you use those criteria?	Based on my experience	No	—
	I am informed concerning the criteria that can help me in discriminating true versus fake news because of:	No	—
	I do not know	No	—

*Note*: The column direction indicates where we find statistically significant higher percentages.

## 4 Discussion

In the current survey, we wanted to understand the criteria that general population uses to judge whether news are true or fake and possible differences in the adoption of such criteria. In general, we found that people referred to mostly use the same criteria for judging news as true or fake; however, their frequency of the adoption changed based on the type of news.

Precisely, regarding the criteria to consider a news as true, people mainly reported to recognize a news as true when the news is composed of consistent pieces of information (i.e., consistency of the content), they perceive the source as a reliable one (i.e., reliability of the source), there is explicit mention to scientific sources in the news (i.e., presence of references to scientific studies and the reliability of the source), the information reported in the news is perceived as verifiable in other sources (i.e., verifiability of the information), and they find confirmation of the news by searching for it in other sources (i.e., confirmation of the news by searching for it). Similarly, people claimed to conclude that a news is fake by relying on the reliability of the source (i.e., reliability of the source), by searching for confirmation of the presented information in other sources (i.e., confirmation of the news by searching for it), if there are references to scientific articles (i.e., presence of references to scientific studies he reliability of the source), by considering the presence of consistent information (i.e., consistency of the content) and the presence of unusual and superfluous details (i.e., unusual and superfluous details). At first sight, our results support the idea that the evaluation of news is driven by specific characteristics of the news itself, such as the quality of details reported and their consistency, and by the source where the news is reported, and these criteria are used both for true and fake news judgments. These results are in line with Ruffo and Semeraro's (2022) results. In this study, the authors investigated people's evaluation process of true and fake news by providing them a pool of true and fake news and by assigning each participant in one of the five experimental conditions, differing in terms of the length of the news (i.e., just the headline or the full article), the presence or not of references of the source, how the news was evaluated by other people (i.e., the number of people who classified the news as true and the number of people who classified the news as fake). In addition, individuals might also decide to fact‐checking the news. They found that the evaluation of true and fake news did not differ such that the length of the news and its source did not help in the discrimination of true and fake news, and there was a social normative influence; hence, participants’ judgments conformed with the ones of other people, and people fact‐checked the news by checking external information that could corroborate the one under evaluation. Our findings seem to concord with these results suggesting that news elaboration and discrimination rely especially on the quality of the information reported and people's perception of the reliability of the source.

However, although the main criteria adopted for true versus fake news were similar, when we statistically compared criteria frequencies, we found interesting differences between true and fake news. As a matter of fact, the statistical comparisons showed that the majority of criteria are used on a higher rate to conclude that a news is true rather than fake, for which we only found higher rates for two criteria (i.e., emotions aroused when reading/listening to the news and sharing of the news by other people). This means that people to judge news as true consider a higher number of characteristics of the news and their source as compared to the elements taken into consideration for fake news judgments. That is, overall, to reach the conclusion that they are dealing with a true news item, individuals reflect on the presence or absence of numerous verbal indicators, such as the consistency, clarity, the amount and type of details (i.e., superfluous and context‐related), the verifiability of the content, as well as information related to the reliability of the source and possible external corroborations. So, it could be speculated that the process conducting to believe news as true is more time‐consuming as well as more cognitive demanding due to the multiple characteristics people would search for and use to recognize them. By contrast, the fact that, in general, people reported the use of less criteria to fake news judgments seems to suggest that these judgments are more automatic and intuitive. These data fit with dual‐process models of elaboration information processing (e.g., Chen and Chaiken 1999; Petty and Cacioppo 1986) assuming that information elaboration can be either analytic or intuitive. Consequently, while dealing with news, people process it either by considering several cues in the news or by relying on heuristics, and this depends on the characteristics of the news itself (Bryanov and Vziatysheva 2021). In addition, the datum that people report to use the emotions aroused when reading/listening to the news and the fact that the news was shared by other people underline that fake news judgments are more frequently based on non‐verbal indicators than true news judgments. This could be explained by speculating that, although fake news by definition try to replicate true news in their forms, they present different features in their content, for instance in terms of emotions expressed (e.g., Giachanou, Rosso, and Crestani 2019; Zhou et al. 2020; Zuckerman, DePaulo, and Rosenthal 1981). This speculation is also in accordance with prior research showing that the emotion elicited by true versus fake news is different, such that, in general, fake news convey negative emotion as compared to true news, although it may also depend on the topic of the news (e.g., natural disaster) (Voloch et al., 2024). Consequently, it could be that these features are particularly prominent in the news, and this guides individuals’ judgment. These results are also in line with previous research showing that the spread of fake news is due to the bandwagon effect, that is, the tendency of people to trust others’ opinions and beliefs concerning a public opinion (Schmitt‐Beck 2015; Wang, Yang, and Xi 2018).

Moreover, this pattern of results suggests that, on a certain way, individuals rely on the same criteria used to develop several forensic techniques to discriminate if a statement is truthful or deceptive. Numerous forensic techniques have been developed (e.g., the CBCA, Köhnken 2004; the RM¸ Johnson and Raye 1981; the VA, Nahari, Vrij, and Fisher 2012) so far, and although they oftentimes rely on the investigation of similar verbal characteristics, they are based on different assumptions. That is, the conclusions of truthfulness or deception are reached by considering the presence or absence of specific verbal characteristics differently. Our data do not allow us to clearly understand the individual's alignment in this regard. Indeed, we asked participants only to select the criteria adopted to make their judgments without indicating the weight of each criterium in the participant's decisions. Therefore, we are not able to argue how people are influenced by the selected criteria to reach their judgments.

Furthermore, when we asked participants whether they used the selected criteria regardless of source of information, a large majority of participants reported to do so both for true and fake news judgments. Indeed, almost 90% of individuals declared to use the same criteria to evaluate the authenticity of news coming from different sources, such as television, social media, or newspapers. This means that there are no specific elements associable with different news sources that influence people's judgments. This is interesting in light of the tangible differences among sources. For instance, social media permit to know how many times a given news item has been shared by other people, whereas television does not. These surprising results need to be further investigated considering prior studies showing, for instance, that some characteristics of online sources of information are frequently adopted by people to make their judgments (e.g., Kluck, Schaewitz, and Krämer 2019).

Noteworthy, people reported almost on an equally base to use the selected criteria based on either their prior experience or on a specific education on the matter, both for true and fake news judgments. These results lead to two considerations. On one hand, it seems that a high percentage of people do understand the importance of being adequately informed on this topic, thus educating themselves. As a matter of fact, to the follow‐up questions on how they were educated on news discrimination, almost all people reported to have consulted scientific literature suggesting therefore that people recognize the importance of the impact of wrong discriminations of news, thus being personally involved in searching and reading evidence‐based knowledge. On the other hand, although this is a promising result, almost another half of participants referred to base their judgments on prior experience. This is alarming considering that prior experience does not necessarily reflect evidence‐based judgments. Indeed, several studies have highlighted that people hold numerous misconceptions regarding several cognitive processes (e.g., memory) and individuals’ abilities, such as deception detection ability (e.g., Bogaard and Meijer, 2018), and these misconceptions can, in turn, impact people's abilities. For instance, Bogaard and Meijer (2018) surveyed a sample of undergraduates’ and police officers about deception detection beliefs and, more precisely, the use of verbal indicators for truthful/deceptive information discrimination. The authors also tested whether people's beliefs affected their deception detection ability. They found that a high percentage of both samples of people hold not‐scientific‐based beliefs on verbal indicators and deception detection and, importantly, that people's beliefs improved or decreased people's ability to distinguish true and false information. In particular, those holding evidence‐based beliefs were more accurate in their discrimination than those holding misconceptions. Hence, our datum suggests the urgent need to divulgate scientific knowledge to educate people and, consequently, prevent fake news endorsement.

## 5 Limitations, Future Directions, and Conclusion

Some caveats of the present study need to be mentioned. First, we wanted to investigate which criteria general population use to consider news as true or fake. We, therefore, proposed multiple choice questions with possible criteria useful for such judgments. However, our options did not inform us concerning how the chosen criterium influences true versus fake judgment. For instance, if the individual reported to use the consistency of details criterium to suppose a news is true, we do not know whether having highly consistent information in the news made the individual prone to conclude the news is true or, rather, the person made such a judgment when they found inconsistent information. In addition, we were interested in Italian population; hence, our results are related to Italian people. Instead, it could be beneficial having future work collecting information in different countries to have information on possible cross‐cultural issues. Moreover, we did not test in our study whether individual differences, such as cognitive and personality traits, drive people in the use of specific criteria for true versus news judgments. So far, several studies have shown that fake news detection is influenced by individual traits (e.g., Bronstein et al. 2019; Calvillo et al. 2021; Pennycook and Rand 2020; Sindermann, Cooper, and Montag 2020; Wolverton & Stevens, 2020). Hence, future investigation could be considered to explore further how individual differences affect true versus fake news judgments. Moreover, we did not ask participants for information concerning how many times they discovered that their judgment of truthfulness versus fakeness of the news was incorrect. This could be another interesting issue to take into account in future investigations. Still, future studies could investigate whether the specific content of the news (e.g., political, medical, and general knowledge content) or the type of fake news may influence which criteria people rely on to make their judgments.

To conclude, our study provides important information on which criteria people adopt to distinguish whether news is true or fake. This information is useful to prevent the acceptance of fake news as true news in the general population. Believing in fake news can have detrimental effects, such as the spread of false information in society, which, in turn, can influence behavioral choices. As such, the education of general population concerning possible ways to distinguish true news from fake news is an important societal issue. Our results can help in designing such educational training by focusing their intervention in disentangling the use of criteria that, based on scientific literature, do not allow individuals to make a better detection of fake information.

## Author Contributions

**Fabiana Battista**: conceptualization, investigation, funding acquisition, writing–original draft, methodology, formal analysis, data curation, project administration. **Tiziana Lanciano**: conceptualization, writing–review and editing. **Antonietta Curci**: conceptualization, writing–review and editing, supervision.

## Conflicts of Interest

The authors declare no conflicts of interest.

### Peer Review

The peer review history for this article is available at https://publons.com/publon/10.1002/brb3.70315.

## Data Availability

The data that support the findings of this study are available from the corresponding author upon reasonable request.
